# Exploration of healthcare workers’ perceptions on occupational risk of HIV transmission at the University of Gondar Hospital, Northwest Ethiopia

**DOI:** 10.1186/1756-0500-5-704

**Published:** 2012-12-29

**Authors:** Getahun Asres Alemie

**Affiliations:** 1 College of Medicine and Health Sciences, University of Gondar, Gonder, Ethiopia

**Keywords:** Healthcare workers’ perception, HIV/AIDS, Occupational risk

## Abstract

**Background:**

HIV/AIDS has several means of transmission. Exposure to blood and other body fluids is a very important means of transmission. Healthcare workers are exposed to this disease mainly due to the nature of their work. This is an exploration of the perceptions of healthcare workers of the University of Gondar Hospital.

**Methods:**

Based on purposive sampling seven healthcare workers were selected from different departments in the hospital so that they could reflect on their perceptions. The selected healthcare workers were asked about the risks related to their work, their experience of HIV related hazards and their general views on the transmission of HIV. The main themes were identified for analysis and the views were summarized under the themes.

**Results:**

All the respondents were aware of the risk of acquiring HIV in healthcare settings. Some had experienced accidents that made them take post-exposure prophylaxis, and most witnessed accidents like needle-stick injuries to their colleagues. They also expressed their feelings that their workplace was not the best place to work at.

**Conclusion:**

Health professionals are well aware of the possibility of HIV transmission associated with their practice. Accidents like needle stick injuries are apparently common; and at the same time, the practice of healthcare workers towards using universal precautions looks poor.

## Background

HIV has many transmission routes the most common of which being sexual intercourse. The other routes of transmission include mother to child transmission, exposure to blood and blood products or contaminated body fluids [1]. Body fluids are encountered during different medical interventions, and are hence responsible for occupation related HIV transmission [2]. Healthcare workers have a 0.3% risk of acquiring HIV following a contaminated needle stick injury [3,4].

Occupational risk of HIV transmission is a major concern for healthcare workers [5]. Some healthcare workers are at a greater risk of acquiring HIV infection than others. These include those who are in close contact with body fluids like surgeons, obstetricians, midwives and laboratory personnel. These people need extra precaution while handling their patients [6].

As a result of its numerous effects, HIV has significantly altered the delivery of healthcare and the lives of people virtually in all communities. The risk of transmission, particularly through needle-stick injuries, continues to be a major concern for all professionals working in healthcare settings [7,8].

This study aimed to examine the perceptions of healthcare workers on work place transmission of HIV and its effects on healthcare.

## Methods

### Design and setting

This is a qualitative study carried out in 2009 at the UoG Hospital which is the biggest teaching referral hospital in Northwest Ethiopia. Among the services rendered by the hospital are surgical operations, care and treatment to patients with HIV/AIDS, delivery service for women, extensive laboratory work, etc.

### Study participants

Study participants were healthcare workers from different service areas of the hospital. The participants were purposively selected by the investigator. First the major service areas of the hospital potentially at a higher risk of work related HIV transmission were identified: maternity ward, medical and surgical wards, and hospital laboratory. A general practitioner, intern doctors, a clinical nurse, a midwife nurse and a laboratory worker were recruited. A total of seven respondents were included. Nobody refused to participate in the study.

### Data collection

The data collection took place in the hospital. Questionnaires with open ended questions were used as the data collection tool. The questions included age and sex of the professionals, their perceived risk of work related HIV transmission and experience of work related accidents. The following are the questions included in the questionnaire:

1. Describe your age, sex and work experience

2. What is your profession? What do you really do in the hospital? List all possible tasks you do.

3. What are the possible occupational hazards related to HIV transmission at your workplace?

4. Have you ever experienced any of the risks you mentioned?

5. Do you think HIV is a common risk in the health workers’ environment? Why?

6. What types of care do you give for HIV infected patients in the hospital?

7. What does the hospital have to do in order to deal with this work related risk of HIV transmission?

8. Do you know someone with needle stick injury accident in your workplace? How did she/he behave during the incident? Explain in detail.

9. How do you approach a health worker with any risk of HIV transmission related to work?

### Analysis

Data was analyzed using thematic approach. The documents containing participants’ responses were read and re-read by the investigator to identify emerging themes. Different ideas were picked during the analysis and these ideas were used to describe similar beliefs and practices, and develop explanations. Data was summarized under the identified themes.

Ethical approval was obtained from the School of Public Health, College of Medicine and Health Sciences, University of Gondar. In addition, official permission was obtained from the hospital before conducting the study. On top of these, the participants were well informed about the purpose of the study, and also knew that data would be collected anonymously and that no information will be shared to a third party mentioning their names. Verbal consent was then obtained from the study participants. Written consent was not done for the exploration is basically a low risk study; the data collection was anonymous and there were no interventions before or after collecting data.

## Results

Out of the seven informants one was female. All were directly involved in handling patients with HIV infection. One of them was a general practitioner engaged in delivering women at the maternity ward of the hospital and doing gynecologic and obstetric procedures. Three others were intern doctors who cared for medical, surgical, gynecologic, obstetric and pediatric patients. Other 2 were nurses: a clinical nurse and a midwife. And there was one laboratory worker.

Five major themes emerged:

1. Work Related Transmission of HIV Infection

All the professionals included in the study had adequate awareness about the transmission of HIV infection at workplace. They had mentioned several means of transmission. Some of them were: injury with sharp materials and needles; exposure to body fluids; and contact with wounds during dressing. The participants believed that the major reasons for a healthcare worker acquiring HIV infection at a work place are all related to one or more of the mentioned means.

2. Experience of Injuries at Workplace

Four of the seven subjects had experienced accidents: needle stick injuries, exposure to blood or other body fluid. Their explanations of the incidents indicated the accidents were frequent. They also witnessed a number of other workers experiencing the accidents, and hence indicating the commonness of the problem in healthcare settings.

"“At workplace, I have experienced needle prick injury and splash of amniotic fluid into my mouth and eyes.” (A 24 year old midwife)"

While describing the emotional reactions of the victims during the accidents they generally expressed their sorrow by citing the observed reactions. The following were the direct quotes from the participants:

"“In my experience I know a person who sustained needle stick injury while drawing blood and was really worried and the reaction he took was to stop his work for about 1 month.” (A 24 year old intern doctor)"

"“I know three laboratory technicians who sustained needle stick injuries and took post exposure prophylaxis. During the incident, one of them, a friend of mine, shouted and immediately burst into tears and he even tried to cut his finger.” (A 26 year old medical laboratory technologist)"

Many of the witnessed injuries/accidents were in fact followed by commencement of post exposure prophylaxis which, however, was mentioned by some to be less practiced. In line with this, professionals were well aware of post exposure prophylaxis despite some problems in getting the service at the hospital.

3. The Need for Protective Materials

All the study participants were worried about the inadequacy of protective materials essential in the prevention of HIV transmission in healthcare settings. This was reflected by the respondents mentioning it as the main reason for why the risk of work related HIV transmission was perceived to be high among healthcare workers. Among the materials mentioned to be inadequate were gloves, gowns, goggles and aprons. Additional interventions were also suggested. For example one of the study participants stated the following:

"“Our hospital has to do the following activities in order to handle work related risk of HIV transmission: giving service to HIV patients in a separate place and taking extra care; training healthcare workers on infection prevention; and organizing a committee that can follow the use of universal precautions in the hospital.” (A 26 year old medical laboratory technologist)"

4. Reasons Why HIV Transmission is a Common Risk among Health Professionals

Many reasons were mentioned by the informants. One of them was that medical interventions were related with blood and other body fluids and the use of sharp materials. The other was many patients were not screened and health professionals were generally less careful while caring for those patients which potentially predisposes them for infection. The presence of poor team work among healthcare workers was also raised as a reason enhancing the transmission of HIV infection. With all the mentioned reasons health workers believed that they had a great risk of acquiring HIV at workplace.

5. Care to HIV Patients

Despite all the risks associated with care for HIV patients all professionals strongly reported that they were in good relation with patients with HIV and they treated them just like the other patients.

## Discussion

This study demonstrated that the possibility of work related HIV transmission was well appreciated by healthcare workers. Many studies support that the risk of acquiring HIV infection at workplace is well recognized, even by lower level healthcare workers [6,9]. The problem actually lies on the possible measures healthcare workers take and the nature of the working environment in terms of preventing the disease among healthcare workers [5].

Looking into the results of other studies, it is clear that many healthcare workers sustain accidents like needle stick injuries at workplace [3,10,11]. This study also suggests that similar situation exists at the University of Gondar Hospital. Together with the possibility of acquiring HIV at workplace, there is also an additional psychological impact on the healthcare workers. This has been shown by the reaction of healthcare workers to such incidents at workplace in general [12,13].

Though this study does not point out the possible impacts of workplace risk of HIV transmission on the quality of patient care, it is believed that risk of acquiring HIV at workplace contributes to the poor healthcare provided to some of the patients. Many studies show that exposure to HIV/AIDS in healthcare settings result in negative effects on the quality of care for people living with HIV [12,14,15].

In addition to what is discussed above, this study stresses on the complaint of healthcare providers regarding the availability of protective materials. Universal precautions and protective materials are the most important defense healthcare workers have against HIV transmission at workplace [6]. Many healthcare institutions still lack the capacity to provide these things to the satisfaction of healthcare workers. This in turn has resulted in fatigue and disappointment among healthcare workers, including dropout from work [5,12]. On the other hand, there is also negligence among health professionals in using universal precautions as to the standard recommendations which adds to the existing problem [9].

Special emphasis has to be given to healthcare workers who work in close contact with blood, blood products and body fluids which are potentially contaminated with HIV [16,17]. Studies suggest that the risk of HIV transmission varies across different fields of medical practice, and hence the need for special emphasis to those with higher risk of transmission of blood borne diseases in general [6,18].

One of the limitations of this study is that only a few health workers have participated in the study and generalizability might be compromised. In addition, the use of a convenience sampling technique while selecting participants is a concern. Moreover, focus group discussion with active interaction of professionals would have indicated many more important issues.

## Conclusion

Health professionals are generally well aware of the risk of acquiring HIV at workplace. Accidents like needle stick injuries are apparently common; and at the same time, the practice of healthcare workers towards using universal precautions looks poor.

### Recommendation

Training healthcare workers on universal precautions is still very vital to reduce the occupational risk of HIV transmission. Healthcare institutions should also be equipped with adequate protective materials. Further investigations are also recommended to strengthen the data.

## Availability of supporting data

There are no supporting data presented and linked with this study.

## Competing interests

There are no competing interests.

## Authors’ contributions

The author developed the proposal, collected the data, analyzed the data and prepared the manuscript and sent it for publication.

## References

[B1] Fauci AS Kasper Human immunodeficiency virus disease: AIDS and related disorders Harrison’s Principles of Internal Medicine 2005 16 USA: McGrawHill Medical Publishing Division 1079

[B2] Tolle MA Schwarzwald HL Postexposure Prophylaxis against human immunodeficiency virus. Am Fam Physician 2010 82 2 161 166 20642270

[B3] Hadadi A Afhami S Karbakhsh M Esmailpour N Occupational exposure to body fluids among healthcare workers: a report from Iran. Singapore Med J 2008 49 6 492 496 18581025

[B4] Trim JC Elliott TSJ A review of sharps injuries and preventative strategies. J Hosp Infect 2003 53 237 242 10.1053/jhin.2002.1378 12660120

[B5] Reda AA Fisseha S Mengistie B Vandeweerd JM Standard precautions: occupational exposure and behaviour of healthcare workers in Ethiopia. PLoS One 5 12 e14420 10.1371/journal.pone.0014420 21203449 PMC3009714

[B6] Nwankwo TO Aniebue UU Percutaneous injuries and accidental blood exposure in surgical residents: awareness and use of Prophylaxis in relation to HIV. Niger J Clin Pract 2011 14 1 34 37 10.4103/1119-3077.79237 21493989

[B7] Taylor KM Eakin JM Skinner HA Kelner M Shapiro M Physicians’ perception of personal risk of HIV infection and AIDS through occupational exposure. Can Med Assoc 1990 143 6 493 500 PMC1452290 2207904

[B8] Emmanuel EJ Do physicians have an obligation to treat patients with AIDS? N Engl J Med 1988 318 1686 1690 10.1056/NEJM198806233182511 3374540

[B9] Parmeggiani C Abbate R Marinelli P Angelillo IF Healthcare workers and healthcare associated infections: knowledge. attitudes and behaviour in emergency departments in Italy. BMC Infect Dis 2010 10 35 10.1186/1471-2334-10-35 20178573 PMC2848042

[B10] Chacko J Isaac R Percutaneous injuries among medical interns and their knowledge and practice of post-exposure Prophylaxis for HIV. Indian J Public Health 2007 51 2 127 129 18240478

[B11] Gupta A Anand S Sastry J Krisagar A Basavaraj A Bhat SM Gupte N Bollinger RC Kakrani AL High risk for occupational exposure to HIV and utilization of post-exposure Prophylaxis in a teaching hospital in Pune, India. BMC Infect Dis 2008 8 142 10.1186/1471-2334-8-142 18939992 PMC2588594

[B12] Lin C Wu Z Wu S Jia M Occupational exposure to HIV among healthcare providers: a qualitative study in Yunnan, China. J Int Assoc Physicians AIDS Care (Chic) 2008 7 1 35 41 17641135 10.1177/1545109707302089 PMC2791920

[B13] Bennett L The experience of nurses working with hospitalized AIDS patients. Aust J Soc Issues 1992 27 125 143

[B14] Adebamowo CA Ezeome ER Ajuwon JA Ogundiran TO Survey of the knowledge. attitude and practice of Nigerian surgery trainees to HIV-infected persons and AIDS patients. BMC Surg 2002 2 7 10.1186/1471-2482-2-7 12201903 PMC126215

[B15] Kelly JA Stigmatization of AIDS patients by Physicians Am J Public Health 1987 77 789 791 10.2105/AJPH.77.7.789 3592030 PMC1647216

[B16] Wicker S Cinatl J Berger A Doerr HD Gottschalk R Rabenau HF Determination of risk of infection with blood-borne pathogens following a needle stick injury in hospital workers. Ann Occup Hyg 2008 52 7 615 622 10.1093/annhyg/men044 18664514

[B17] Agustian D Yusnita S Susanto H Sukandar H Schryver AD Meheus A An estimation of the occupational risk of HBV, HCV and HIV infection among Indonesian healthcare workers. Acta Med Indones-Indones J Intern Med 2009 41 Supplement 1 33 37 19920296

[B18] Lowenfels AB Wormser GP Jain R Frequency of puncture injuries in Surgeons and estimated risk of HIV infection. Arch Surg 1991 126 789 790 2818181 10.1001/archsurg.1989.01410110038007

